# Supplementary material for: A Humanized Yeast Phenomic Model of Deoxycytidine Kinase to Predict Genetic Buffering of Nucleoside Analog Cytotoxicity
Source: Genes (Basel). 2019 Sep 30;10(10):770. doi: 10.3390/genes10100770 (PMC6826991; doi:10.3390/genes10100770)
Supplement: Supplementary file 1 [file genes-10-00770-s001.zip › Additional_Files/Additional_File7_GOTermHeatmaps.zip!/Additional_File7_GOTermHeatmaps/structural_molecule_activity.pdf]

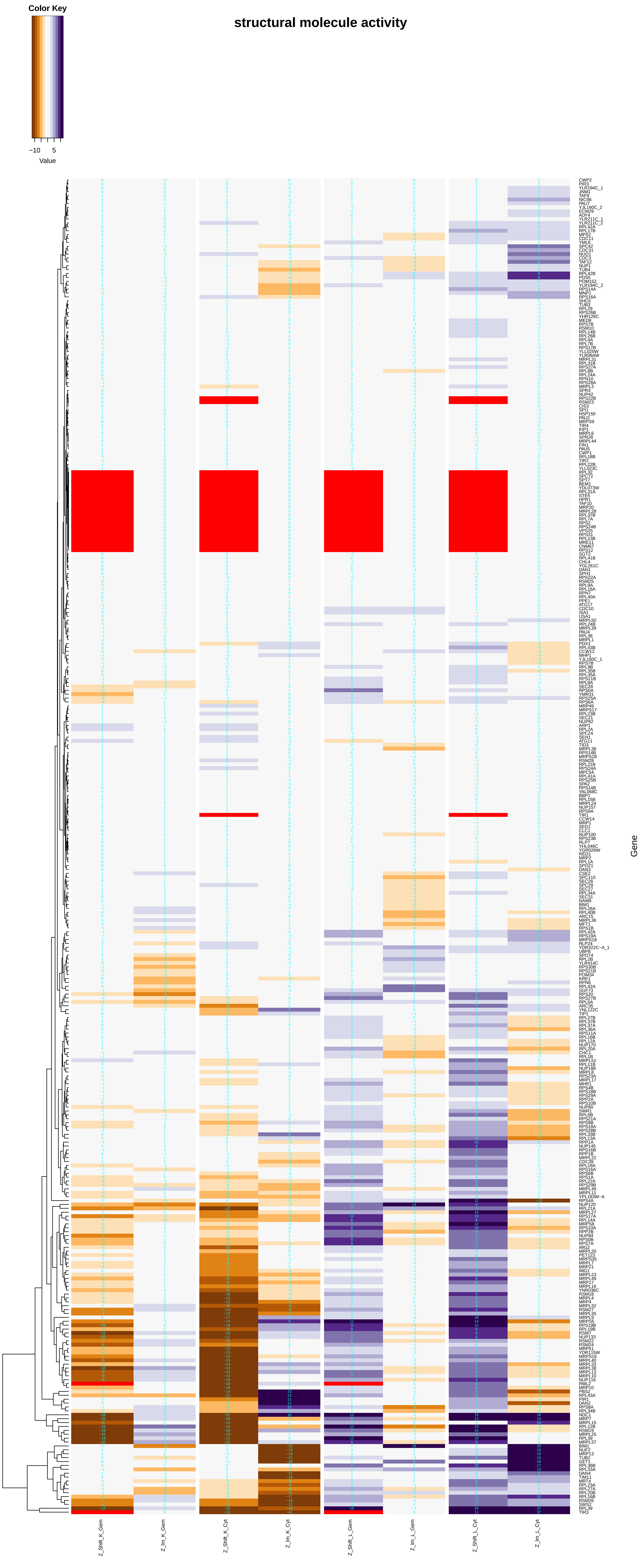
structural molecule activity
Color Key
−10
5
Value
Gene
Z_Shift_K_Gem
Z_Im_K_Gem
Z_Shift_K_Cyt
Z_Im_K_Cyt
Z_Shift_L_Gem
Z_Im_L_Gem
Z_Shift_L_Cyt
Z_Im_L_Cyt

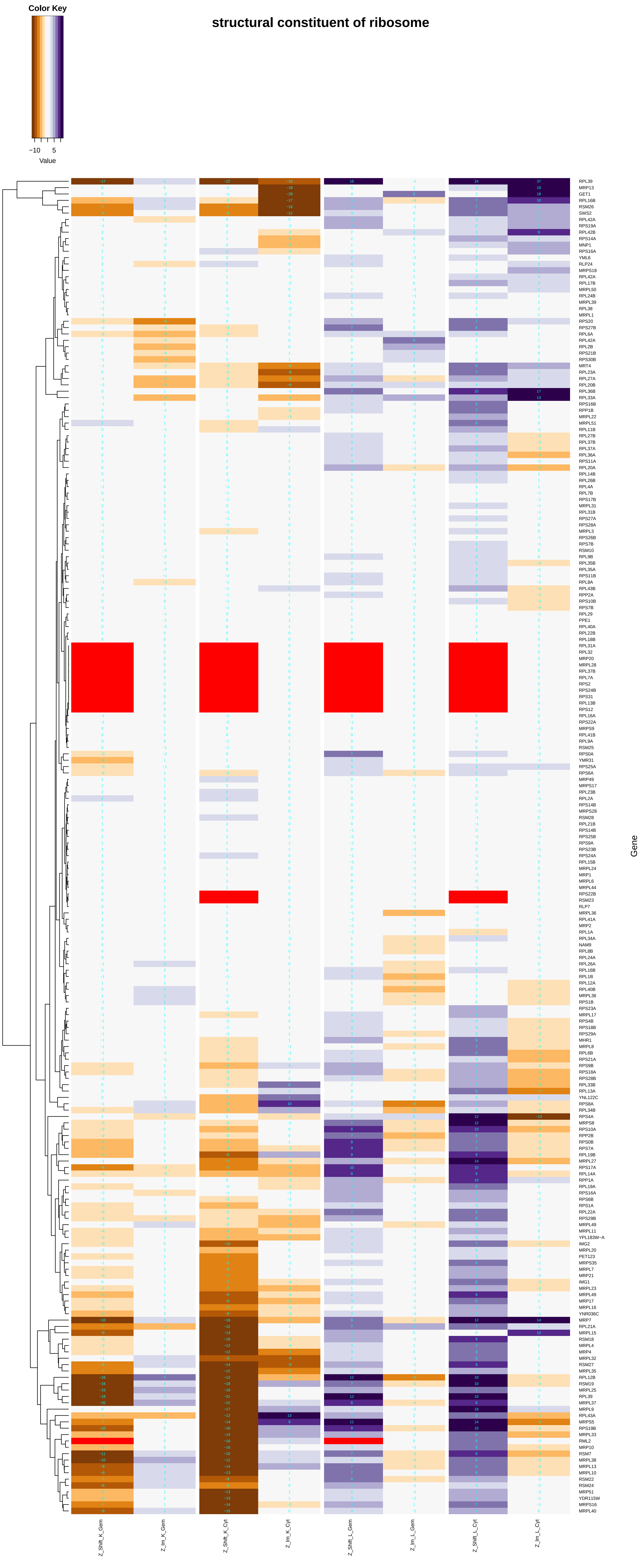

structural constituent of ribosome
Color Key
−10
5
Value
Gene
Z_Shift_K_Gem
Z_Im_K_Gem
Z_Shift_K_Cyt
Z_Im_K_Cyt
Z_Shift_L_Gem
Z_Im_L_Gem
Z_Shift_L_Cyt
Z_Im_L_Cyt
RPL39
MRP13
GET1
RPL16B
RSM26
SWS2
RPL42A
RPS19A
RPL42B
RPS14A
MNP1
RPS16A
YML6
RLP24
MRPS18
RPL42A
RPL17B
MRPL50
RPL24B
MRPL39
RPL38
MRPL1
RPS20
RPS27B
RPL6A
RPL42A
RPL2B
RPS21B
RPS30B
MRT4
RPL23A
RPL27A
RPL20B
RPL36B
RPL33A
RPS16B
RPP1B
MRPL22
MRPL51
RPL11B
RPL27B
RPL37B
RPL37A
RPL36A
RPS11A
RPL20A
RPL14B
RPL26B
RPL4A
RPL7B
RPS17B
MRPL31
RPL31B
RPS27A
RPS28A
MRPL3
RPS26B
RPS7B
RSM10
RPL9B
RPL35B
RPL35A
RPS11B
RPL8A
RPL43B
RPP2A
RPS10B
RPS7B
RPL29
PPE1
RPL40A
RPL22B
RPL18B
RPL31A
RPL32
MRP20
MRPL28
RPL37B
RPL7A
RPS2
RPS24B
RPS31
RPL13B
RPS12
RPL16A
RPS22A
MRPS9
RPL41B
RPL9A
RSM25
RPS0A
YMR31
RPS25A
RPS6A
MRP49
MRPS17
RPL23B
RPL2A
RPS14B
MRPS28
RSM28
RPL21B
RPS14B
RPS25B
RPS9A
RPS23B
RPS24A
RPL15B
MRPL24
MRP1
MRPL6
MRPL44
RPS22B
RSM23
RLP7
MRPL36
RPL41A
MRP2
RPL1A
RPL34A
NAM9
RPL8B
RPL24A
RPL26A
RPL16B
RPL1B
RPL12A
RPL40B
MRPL36
RPS1B
RPS23A
MRPL17
RPS4B
RPS18B
RPS29A
MHR1
MRPL8
RPL6B
RPS21A
RPS9B
RPS18A
RPS28B
RPL33B
RPL13A
YNL122C
RPS8A
RPL34B
RPS4A
MRPS8
RPS10A
RPP2B
RPS0B
RPS7A
RPL19B
MRPL27
RPS17A
RPL14A
RPP1A
RPL19A
RPS16A
RPS6B
RPS1A
RPL22A
RPS29B
MRPL49
MRPL11
YPL183W-A
IMG2
MRPL20
PET123
MRPS35
MRPL7
MRP21
IMG1
MRPL23
MRPL49
MRP17
MRPL16
YNR036C
MRP7
RPL21A
MRPL15
RSM18
MRPL4
MRP4
MRPL32
RSM27
MRPL35
RPL12B
RSM19
MRPL25
RPL39
MRPL37
MRPL9
RPL43A
MRPS5
RPS19B
MRPL33
RML2
MRP10
RSM7
MRPL38
MRPL13
MRPL10
RSM22
RSM24
MRP51
YDR115W
MRPS16
MRPL40

structural constituent of cell wall

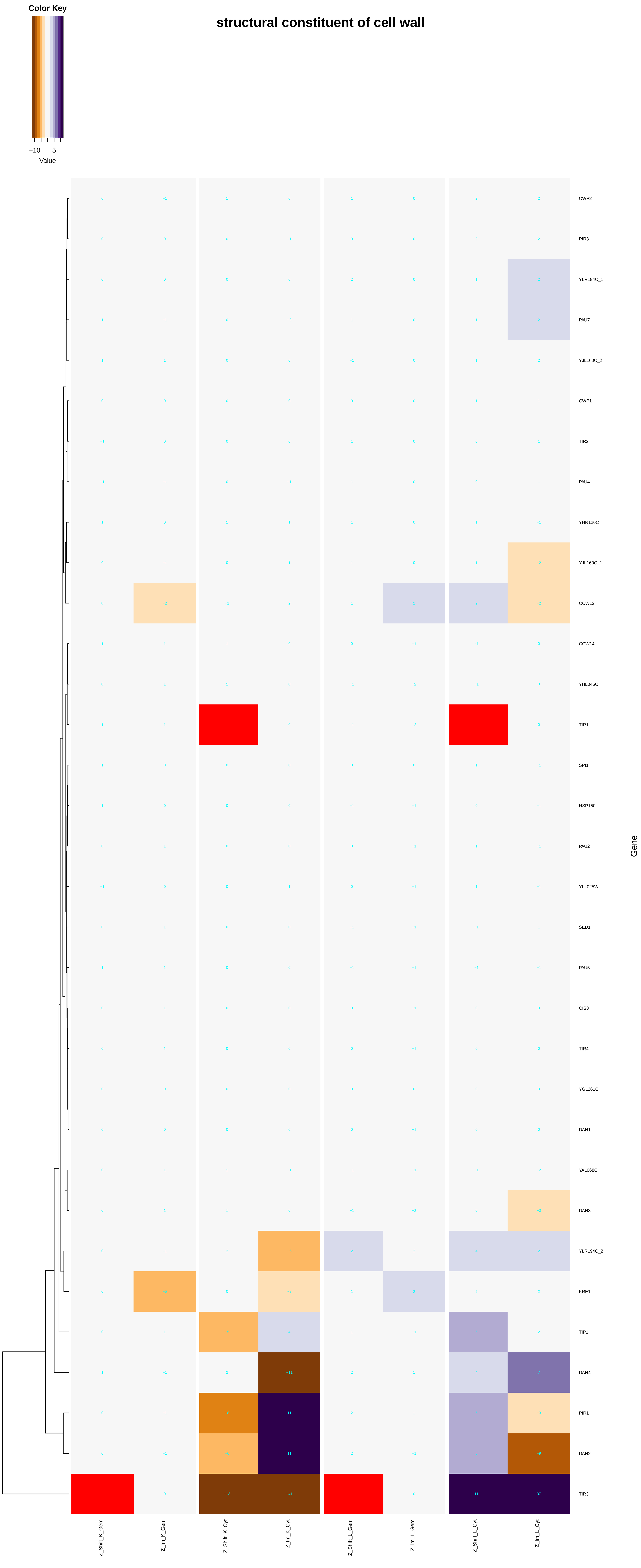

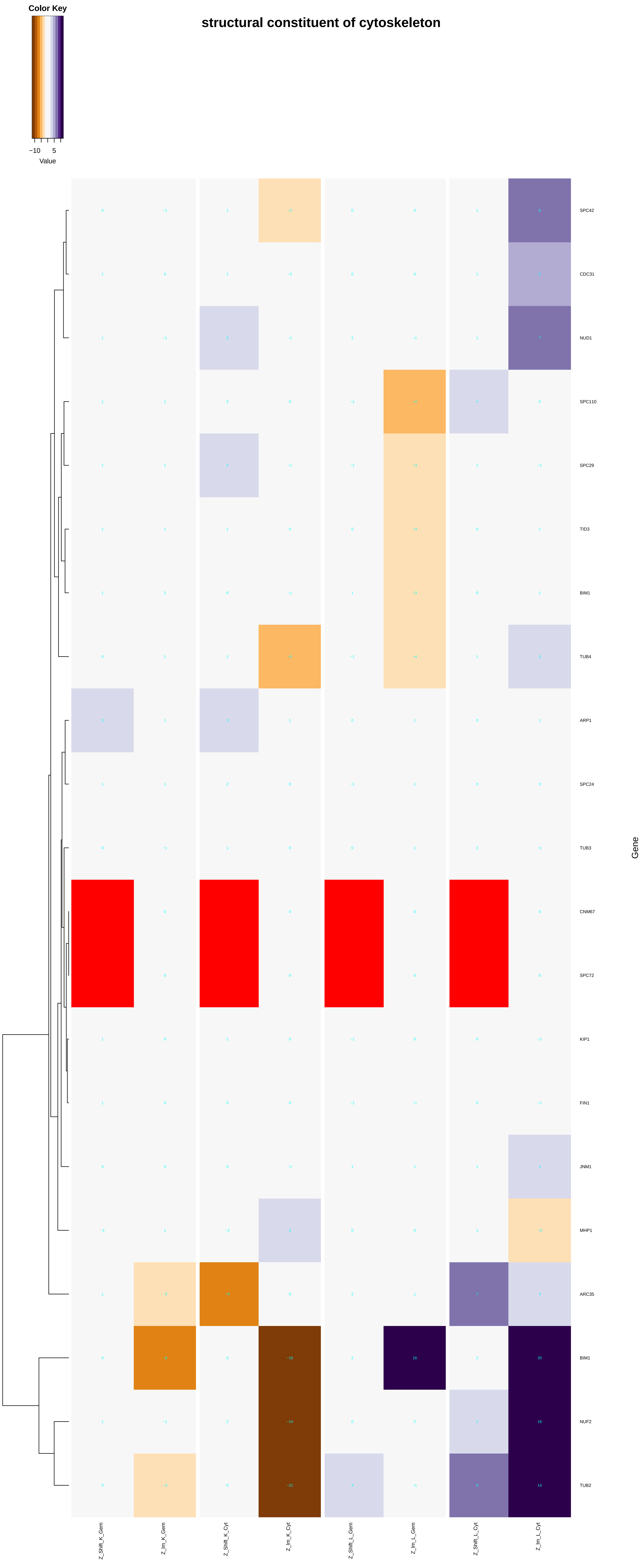
structural constituent of cytoskeleton
Color Key
−10
5
Value
SPC42
CDC31
NUD1
SPC110
SPC29
TID3
BIM1
TUB4
ARP1
SPC24
TUB3
CNM67
SPC72
KIP1
FIN1
JNM1
MHP1
ARC35
BIM1
NUF2
TUB2
Gene
Z_Shift_K_Gem
Z_Im_K_Gem
Z_Shift_K_Cyt
Z_Im_K_Cyt
Z_Shift_L_Gem
Z_Im_L_Gem
Z_Shift_L_Cyt
Z_Im_L_Cyt

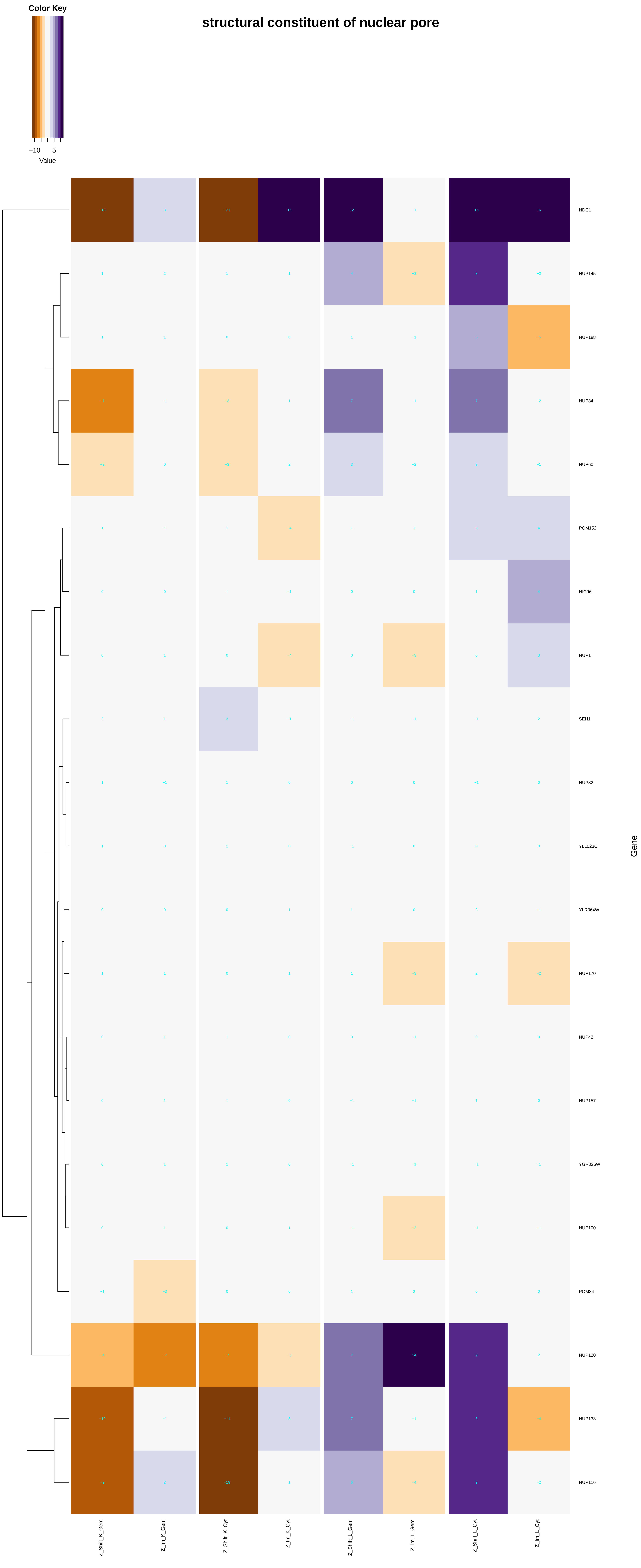

Color Key
−10
5
Value
structural constituent of nuclear pore
NDC1
NUP145
NUP188
NUP84
NUP60
POM152
NIC96
NUP1
SEH1
NUP82
YLL023C
YLR064W
NUP170
NUP42
NUP157
YGR026W
NUP100
POM34
NUP120
NUP133
NUP116
Gene
Z_Shift_K_Gem
Z_Im_K_Gem
Z_Shift_K_Cyt
Z_Im_K_Cyt
Z_Shift_L_Gem
Z_Im_L_Gem
Z_Shift_L_Cyt
Z_Im_L_Cyt

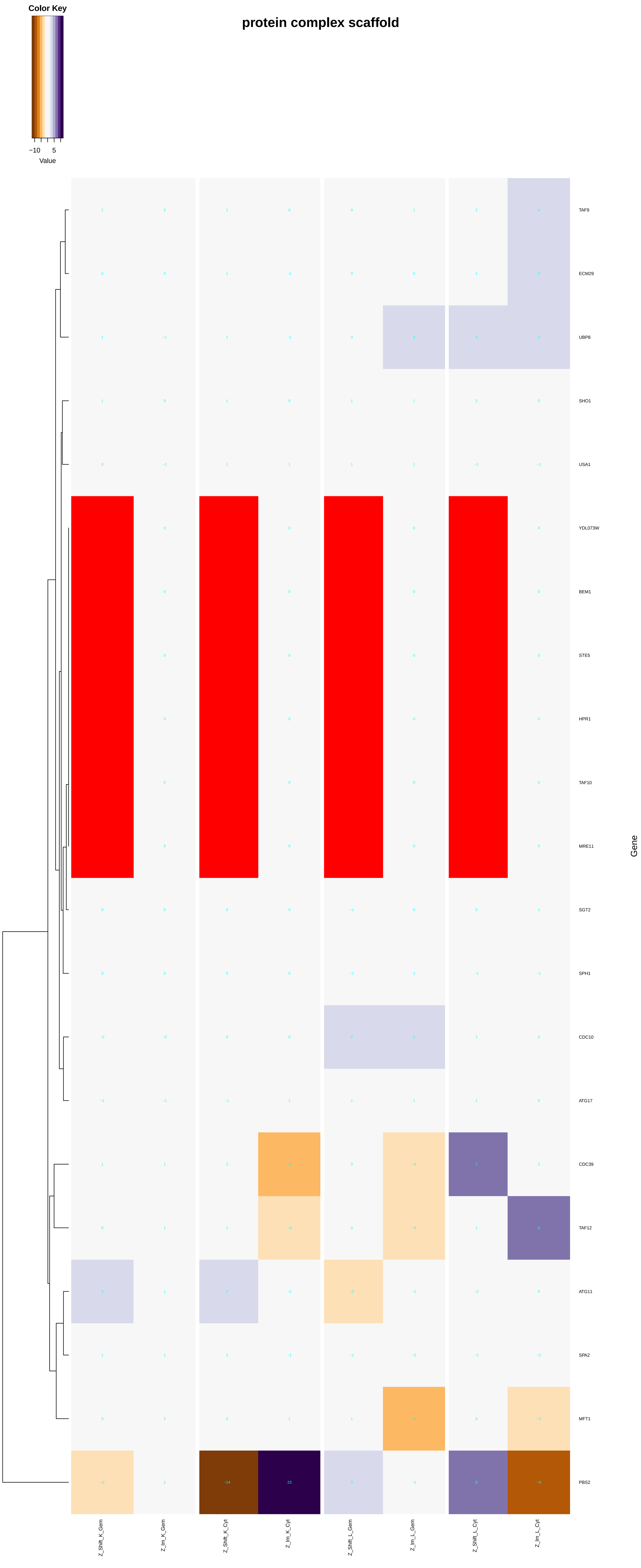
protein complex scaffold
Color Key
−10
5
Value
TAF9
ECM29
UBP8
SHO1
USA1
YDL073W
BEM1
STE5
HPR1
TAF10
MRE11
SGT2
SPH1
CDC10
ATG17
CDC39
TAF12
ATG11
SPA2
MFT1
PBS2
Gene
Z_Shift_K_Gem
Z_Im_K_Gem
Z_Shift_K_Cyt
Z_Im_K_Cyt
Z_Shift_L_Gem
Z_Im_L_Gem
Z_Shift_L_Cyt
Z_Im_L_Cyt

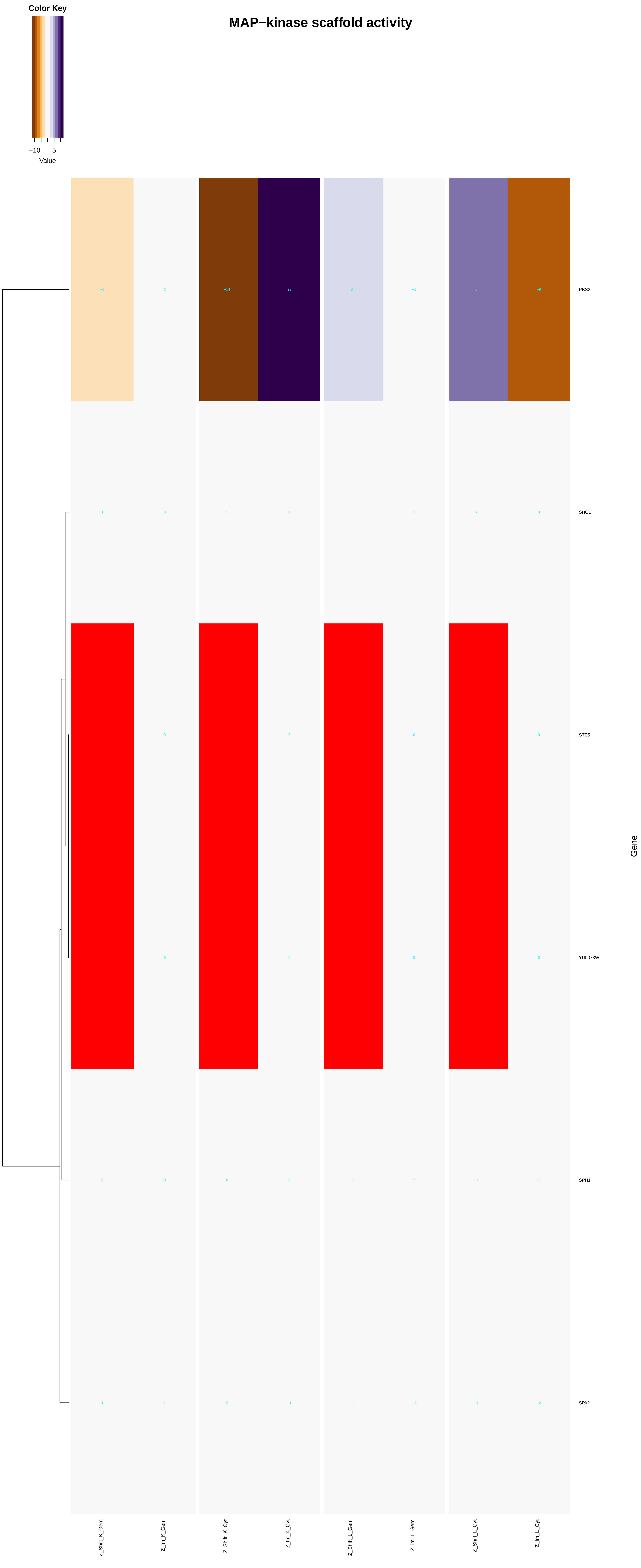
MAP−kinase scaffold activity
Color Key
−10
5
Value
PBS2
SHO1
STE5
YDL073W
SPH1
SPA2
Gene
Z_Shift_K_Gem
Z_Im_K_Gem
Z_Shift_K_Cyt
Z_Im_K_Cyt
Z_Shift_L_Gem
Z_Im_L_Gem
Z_Shift_L_Cyt
Z_Im_L_Cyt
